# A Variable Neighborhood Walksat-Based Algorithm for MAX-SAT Problems

**DOI:** 10.1155/2014/798323

**Published:** 2014-08-06

**Authors:** Noureddine Bouhmala

**Affiliations:** Department of Maritime Technology and Innovation, Buskerud and Vestfold University, Norway

## Abstract

The simplicity of the maximum satisfiability problem (MAX-SAT) combined with its applicability in many areas of artificial intelligence and computing science made it one of the fundamental optimization problems. This NP-complete problem refers to the task of finding a variable assignment that satisfies the maximum number of clauses (or the sum of weights of satisfied clauses) in a Boolean formula. The Walksat algorithm is considered to be the main skeleton underlying almost all local search algorithms for MAX-SAT. Most local search algorithms including Walksat rely on the 1-flip neighborhood structure. This paper introduces a variable neighborhood walksat-based algorithm. The neighborhood structure can be combined easily using any local search algorithm. Its effectiveness is compared with existing algorithms using 1-flip neighborhood structure and solvers such as CCLS and Optimax from the eighth MAX-SAT evaluation.

## 1. Introduction

Many optimization algorithms have been developed for successfully solving a wide range of optimization problems. Although these techniques have demonstrated excellent search capabilities for solving small or medium sized optimization problems, they still encounter serious challenges when applied to solving large scale optimization problems, that is, problems with several hundreds to thousands of variables. How well optimization algorithms handle this sort of real world large scale optimization problems still remains an open question for various optimization problems including MAX-SAT. MAX-SAT is a widely used modeling framework for solving various combinatorial problems. Many important applications can be naturally expressed as MAX-SAT [1]. Examples include routing [2], scheduling [3], model-checking [4] of finite state systems, design debugging [5], AI planning [6], and electronic markets [7]. Interested readers may refer to [8–10] for more details. Efficient methods that can solve large and hard instances of MAX-SAT are eagerly sought. Due to their combinatorial explosion nature, large and complex MAX-SAT problems are hard to solve using systematic algorithms based on branch and bound techniques [11]. One way to overcome the combinatorial explosion is to give up completeness. Stochastic local search algorithms (SLS) are techniques which use this strategy and gained popularity in both worlds whether it is discrete or continuous due to their conceptual simplicity and good performance. The Walksat algorithm [12] is considered to be the main skeleton underlying almost all SLS algorithms for MAX-SAT. It works by assigning all the variables a random truth assignment and then tries to refine the assignment according to a selected heuristic until the CNF formula evaluates to true. The heuristic used for varying the truth assignment defines the variant of Walksat. All variants share the common behavior of exploiting the standard 1-flip neighborhood structure for which two truth value assignments are neighbors if they differ in the truth value of exactly one variable. The critical issue in the design of a neighborhood search strategy is the choice of the neighborhood structure, that is, the manner in which the neighborhood is defined. Larger neighborhood yields better local optima but the computational effort spent to search the neighborhood increases exponentially *O*(*n* ^*k*^) where *k* is the cardinality of neighborhood (i.e., the number of variables to be flipped in order to move from the current solution *s*_*i*_ to a neighboring solution *s*_*j*_) and *n* is the number of variables [13].

In this paper a variable neighborhood Walksat-based algorithm is introduced for MAX-SAT. The key feature of this algorithm aims at identifying improved neighbor solutions without explicitly enumerating and evaluating all the neighbors in the neighborhood. The strategy involves looking at the search as a process evolving from a *k*-flip neighborhood to the standard 1-flip neighborhood-based structure in order to achieve a tactical interplay between diversification (i.e., the ability to explore many different regions of the search space) and intensification (i.e., the ability to obtain high quality solutions within those regions). The authors in [14] discuss the latest design of hybrid approaches in order to find an adequate balance between diversification and intensification.

The rest of the paper is organized as follows. A definition of MAX-SAT is given in Section 2.  Section 3 provides a short survey of methods used to solve MAX-SAT.  Section 4 explains the Walksat algorithm.  Section 5 introduces the variable neighborhood Walksat-Based algorithm and the experimental results. Finally, conclusions are drawn in Section 6.

## 2. The Maximum Satisfiability Problem

Generally, the satisfiability problem (SAT) which is known to be NP-complete [15] is defined as follows. Given is a propositional formula Φ consisting of a set of *N* variables usually represented in CNF (conjunctive normal form). In CNF, the formula is represented as a conjunction of clauses written as Φ = *C*_1_∧*C*_2_∧*C*_3_∧⋯*C*_*M*_, with *M* being the number of clauses. A clause *C*_*i*_(*x*) is a disjunction of literals and a literal is a variable or its negation. As a simple example, let Φ(*x*) be the following formula containing 4 variables and 3 clauses:

(1) $$\begin{array}{c} \Phi \left(x\right)=\left(x1\vee \neg x4\right)\wedge \left(\neg x1\vee x3\right)\wedge \left(\neg x1\vee x4\vee x2\right). \end{array}$$

The task is to determine whether there exists an assignment of values to the variables under which Φ(*x*) evaluates to true. Such an assignment, if it exists, is called a satisfying assignment for Φ, and Φ is called satisfiable. Otherwise, Φ is said to be unsatisfiable. There exist two important variations of the MAX-SAT problem. The weighted MAX-SAT problem is the MAX-SAT problem in which each clause is assigned a positive weight. The goal of the problem is to maximize the sum of weights of satisfied clauses. The unweighted MAX-SAT problem is the MAX-SAT problem in which all the weights are equal to 1 and the goal is to maximize the number of satisfied clauses. In this paper, the focus is restricted to formulas in which all the weights are equal to 1 (i.e., unweighted MAX-SAT).

## 3. Short Survey of SLS for MAX-SAT

Stochastic local search algorithms [16] are based on what is perhaps the oldest optimization method, trial and error. Typically, they start with an initial assignment of values to variables randomly or heuristically generated. During each iteration, a new solution is selected from the neighborhood of the current one by performing a move. Choosing a good neighborhood and a method for searching is usually guided by intuition, because very little theory is available as a guide. All the methods usually differ from each other in the criteria used to flip the chosen variable. One of the earliest local searches for solving SAT is GSAT [17]. The GSAT algorithm operates by changing a complete assignment of variables into one in which the maximum possible number of clauses is satisfied by changing the value of a single variable. Another widely used variant of GSAT is the Walksat based on a two-stage selection mechanism which is originally introduced in [12]. Several state-of-the-art local search algorithms are enhanced versions of GSAT and Walksat algorithms [18–20]. As the quality of the solution improves when larger neighborhood is used, the work proposed in [13] uses restricted 2- and 3-flip neighborhoods and better performance has been achieved compared to the 1-flip neighborhood for structured problems. Clause weighting based SLS algorithms [21, 22] have been proposed to solve SAT and MAX-SAT problems. The key idea is to associate the clauses of the given CNF formula with weights. Although these clause weighting SLS algorithms differ in the manner clause weights should be updated (probabilistic or deterministic), they all choose to increase the weights of all the unsatisfied clauses as soon as a local minimum is encountered. Numerous other methods such as Simulated Annealing [23], Evolutionary Algorithms [24, 25], Scatter Search [26], Greedy Randomized Adaptive Search Procedures [27], and guided local search [28] have also been developed. Lacking the theoretical guidelines while being stochastic in nature, the deployment of several SLS involves extensive experiments to find the optimal noise or walk probability settings. To avoid manual parameter tuning, new methods have been designed to automatically adapt parameter settings during the search [29, 30] and results have shown their effectiveness for a wide range of problems. The work conducted in [31] introduced Learning Automata (LA) as a mechanism for enhancing SLS based SAT solvers, thus laying the foundation for novel LA-based SAT solvers. A new strategy based on an automatic procedure for integrating selected components from various existing solvers has been devised in order to build new efficient algorithms that draw the strengths of multiple algorithms [32, 33]. The work conducted in [34] proposed an adaptive memory based local search algorithm that exploits various strategies in order to guide the search to achieve a suitable tradeoff between intensification and diversification. The computational results show that it competes favorably with some state-of-the-art MAX-SAT solvers. Finally, new solvers have emerged based on a new diversification scheme to prevent cycling [35–37].

## 4. Walksat/SKC Algorithm

In this section, the Walksat/SKC (WS) algorithm originally introduced in [12] which constitutes the chosen local search that will be combined with systematic changes of neighborhood is shown in Algorithm 1.

The algorithm starts with a random assignment (line 3). Thereafter, a random unsatisfied clause is selected (line 5). If there exists a variable belonging to the selected clause with break count equal to zero (line 6), this variable is flipped; otherwise a random variable (line 8) or the variable with minimal break count (line 10) is selected with a certain probability (noise probability: line 7). The break count of a variable is defined as the number of clauses that would be unsatisfied by flipping the chosen variable. It turns out that the choice of unsatisfied clauses, combined with the randomness in the selection of variables, can enable Walksat to avoid local minima and to better explore the search space. The flips are repeated until a preset value of the maximum number of flips is reached (MAX-FLIPS) and this phase is repeated as needed up to MAX-TRIES times.

## 5. The Algorithm

The main difference between metaheuristics relies in the way neighborhood structures are defined and explored. Some metaheuristics work only with a single neighborhood structure. Others, such as numerous variants of variable neighborhood search, operate on a set of different neighborhood structures. Variable neighborhood search (VNS for short) [38–40] aims at finding a tactical interplay between diversification and intensification [16] to overcome local optimality using a combination of a local search and systematic changes of neighborhood. Diversification refers to the ability to explore many different regions of the search space, whereas intensification refers to the ability to obtain high quality solutions within those regions. The basic VNS starts by selecting a finite set of predefined neighborhood structures that will be used during the search. Let *N*_*k*_  (*k* = 1,2,…, *k*_max⁡_) denote the selected set and let *N*_*k*_(*x*) denote the set of solutions in the *k*th neighborhood of *x*. Let *S*_start_ denote the initial solution. VNS starts by generating a random solution *S*_rand_ from the neighborhood *N*_1_(*S*_rand_) ∈ *N*_1_(*S*_start_). Let *S*_new_ denote the reached local optimum when a local search is used with *S*_rand_ as input. If *S*_new_ is better compared to *S*_rand_, the solution is updated and a new round of local search with a random solution from *N*_1_(*S*_new_) is performed. If the test fails, VNS moves to the next neighborhood. The effectiveness of VNS is strongly affected by the ordering in which a given type of neighborhood is considered [41]. Bearing this concept in mind, it is obvious that the application order of the neighborhood structures is crucial for the performance of VNS. Most of the work published earlier on VNS starts from the first neighborhood and moves on to higher neighborhoods without controlling and adapting the ordering of neighborhood structures. Few research articles have begun to search for strategies to dynamically move from one neighborhood to another based on some benefit metrics.  Algorithm 2 shows the details of the variable neighborhood Walksat-based Algorithm which consists of two phases.

*(i) Phase 1.* Let *P* denote the set of variables of the problem to be solved. The first phase of the algorithm consists in constructing a set of neighborhoods satisfying the following property: *N*_1_(*x*) ⊂ *N*_2_(*x*) ⊂ ⋯*N*_*k*_max⁡__(*x*). The starting neighborhood with *k* = 0 consists of a move based on the flip of a single variable. A flip means assigning the opposite state to a variable (i.e., change True → False  or  False → True). The first neighborhood *N*_1_ is constructed from *P* by merging variables. The merging procedure is computed using a randomized algorithm. The variables are visited in a random order. If a variable *l*_*i*_ has not been matched yet, then a randomly unmatched variable *l*_*j*_ is selected and a new variable *l*_*k*_ (a cluster) consisting of the two variables *l*_*i*_ and *l*_*j*_ is created. The set *N*_1_ consists of the move based on flipping predefined clusters each having 2^1^ variables. The new formed clusters are used to define a new and larger neighborhood *N*_2_ and recursively iterate the process until the desired number of neighborhoods (*k*_max⁡_) is reached (lines 3, 4, and 5 of Algorithm 1). Thereafter, a random solution is generated from the largest neighborhood (*N*_*k*_max⁡__) (line 2 of Algorithm 2). The random solution consists in assigning True or False to each cluster and all the literals within that cluster will get the same state.

**Figure 1:**
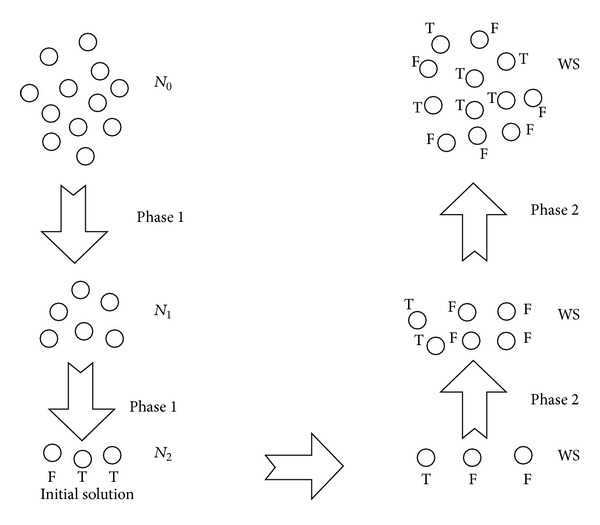
Example illustrating the different phases of the algorithm.

*(ii) Phase 2*. The second phase which is the most crucial aims at selecting the different neighborhoods according to some strategy for the effectiveness of the search process. The strategy adopted in this work is to let VNS start the search process from the largest neighborhood *N*_*k*_max⁡__ and continue to move towards smaller neighborhood structures (lines 7, 8, 9, 10, and 11 of Algorithm 2). The motivation behind this strategy is that the order in which the neighborhood structures have been selected offers a better mechanism for performing diversification and intensification. The largest neighborhood *N*_max⁡_ allows WS to view any cluster of variables as a single entity leading the search to become guided in faraway regions of the solution space and restricted to only those configurations in the solution space in which the variables grouped within a cluster are assigned the same value. As the switch from one neighborhood to another implies a decrease in the size of the neighborhood, the search is intensified around solutions from previous neighborhoods in order to reach better ones. Once the search has reached the convergence criterion with respect to neighborhood *N*_*i*_, the assignment reached on that neighborhood must be projected on its parent neighborhood *N*_*i*−1_. The projection algorithm (line 10 of Algorithm 2) is simple; if a cluster *c*_*i*_ ∈ *N*_*m*_ is assigned the value of true, then the merged pair of clusters that it represents, *c*_*j*_, *c*_*k*_ ∈ *N*_*m*−1_, are also assigned the true value. Finally, the algorithm Walksat is applied at the default neighborhood (line 12 of Algorithm 2). This process is graphically illustrated in Figure 1 using an example with 12 variables. During the first phase, a random merging procedure is used to merge randomly the variables in pairs leading to the first neighborhood *N*_1_ consisting of six clusters each of which is composed of 2 variables. The second neighborhood *N*_2_ is constructed in the same manner. The clusters formed at neighborhood *N*_1_ are merged randomly in pairs leading to a new neighborhood *N*_2_ consisting of three clusters each of which is composed of 2 different clusters each having 2 variables. When the construction of the different neighborhoods comes to its end, a random solution is computed at the neighborhood *N*_2_. Each cluster will be assigned a random value (True or False). Thereafter, the heuristic WS is applied at *N*_2_. When WS flips a cluster from True to False at *N*_2_, all the variables within that cluster (2^2^) will get the same value. When WS reaches the convergence criterion, WS is applied to a smaller neighborhood (*N*_1_), where a move made by WS will consist in flipping a cluster which is having 2 variables. The last step consists in applying WS at *N*_0_ where a move made by WS will consist in flipping a single variable. At this neighborhood, one expects that WS has reached the maximum amount of unsatisfied clauses.

**Figure 2:**
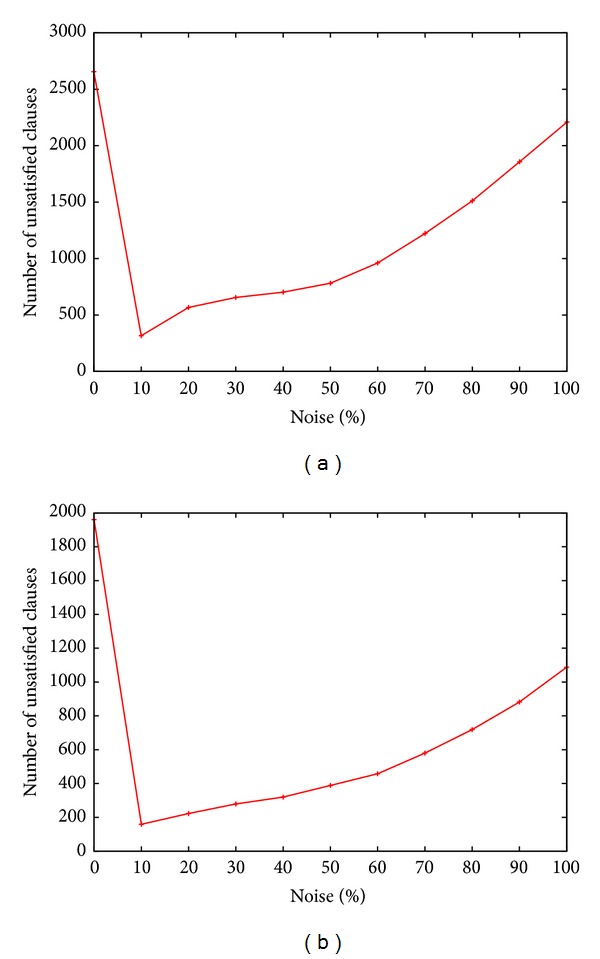
Noise probability versus number of unsatisfied clauses: (a) i2c-master1.dimacs.filtered.cnf: variables = 82429, clauses = 285987, (b) mem-ctrl-debug.dimacs.cnf: variables = 381721, clauses = 505547.

The performance of VNS-WS is evaluated against WS using a set of real industrial problems. This set is taken from the eighth MAX-SAT 2013 organized as an affiliated event of the 16th International Conference on Theory and Applications of Satisfiability Testing (SAT-2013). Due to the randomization nature of both algorithms, each problem instance was run 50 times with a cutoff parameter (max-time) set to 30 minutes. The tests were carried out on a DELL machine with 800 MHz CPU and 2 GB of memory. The code was written in C++ and compiled with the GNU C compiler version 4.6. The following parameters have been fixed experimentally and are listed below:

- *k*_max⁡_: the cardinality of the neighborhood is set such that the number of the formed clusters is 10% of the size of the problem instance (i.e., a problem with 100 literals will lead to *k*_max⁡_ equal to 3).
- WS spends equal amount of time (max-time/*k*_max⁡_) between the different neighborhoods.
- Noise probability: the performance of WS depends highly on the walking probability setting which in turns depends on the class of problems to be solved. The plots in Figures 1 and 2 show four selected tests that reflect the general trend observed on almost all the industrial instances tested. Peak performance with respect to the lowest number of unsatisfied clauses is achieved when the walking probability was set to 10.

### 5.1. Observed Search Trend

**Figure 3:**
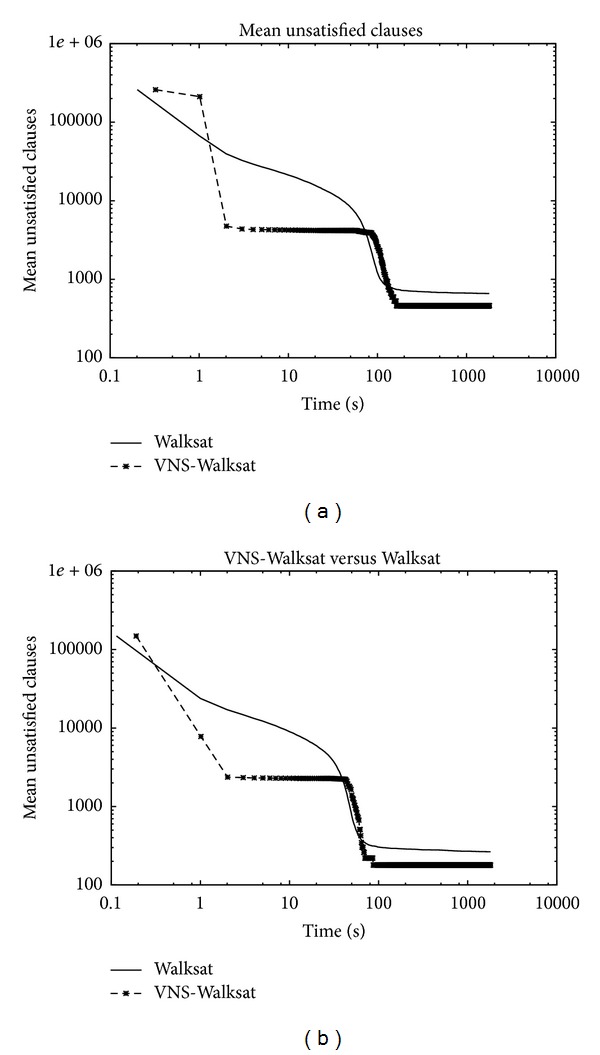
Log-Log Plot: (a) rsdecoder2.dimacs.filtered.cnf: variables = 415480, clauses = 1632526, (b) rsdecoder-fsm1.dimacs.filtered.cnf: variables = 238290, clauses = 936006. Time development for 100 runs in 15 minutes.

**Figure 4:**
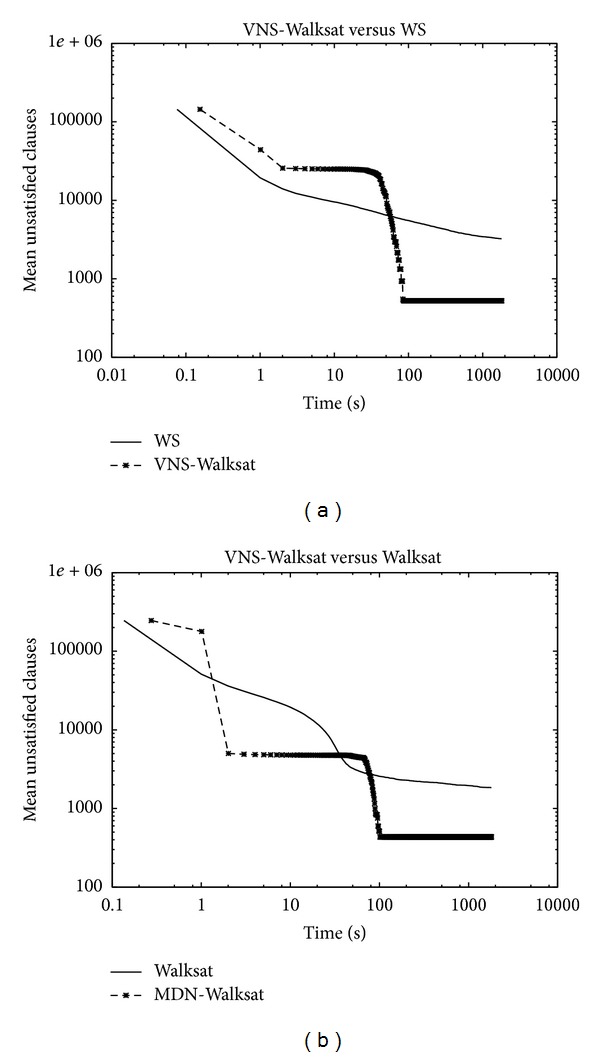
Log-Log Plot: (a) rsdecoder1-blackbox-CSEEblock-problem.dimacs-32.filtered: variables = 277950, clauses = 806460, (b) rsdecoder-multivec1.dimacs.filtered: variables = 394446, clauses = 1626312. Time development for 100 runs in 15 minutes.

Figures 3 and 4 show the evolution of the mean of unsatisfied clauses of both algorithms as a function of time. Both algorithms provide an initial solution of the same quality while showing a crossover in their corresponding curves. During the early phase of the search, the solution quality provided by WS is better compared to VNS-WS. The superiority of WS lasts for a short while before VNS-WS catches up and surpasses WS. Both algorithms were able to decrease the mean number of unsatisfied clauses at a high rate before entering the so-called plateaus region where WS typically encounters a sequence of states that leave the number of unsatisfied clauses unchanged. While WS shows a premature stagnation behavior of the search, VNS-WS was capable of finding neighboring states with fewer unsatisfied clauses, thereby exiting the plateau. VNS-WS shows equal or marginally better asymptotic convergence for small problems compared to WS as the two curves overlay each other closely, while the convergence behavior becomes more distinctive for larger problems. The key behind the efficiency of VNS-WS relies on the variable neighborhood structure. VNS-WS draws its strength from coupling WS across different neighborhoods. This paradigm offers two main advantages which enables WS to become much more powerful. During the improvement phase (i.e., each time WS is called with a different neighborhood), WS applies a local transformation (i.e., a move) within the neighborhood (i.e., the set of solutions that can be reached from the current one) of the current solution to generate a new one. The selected variable neighborhood structure offers a better mechanism for performing diversification and intensification. By allowing WS to view a cluster of variables as a single entity, the search becomes guided and restricted to only those configurations in the solution space in which the variables grouped within a cluster are assigned the same value. The switch from one neighborhood to another implies a decrease in the size of the neighborhood leading the search to explore different regions in the search space, while intensifying the search by exploiting the solutions from previous neighborhoods in order to reach better ones.

### 5.2. Convergence Speed

**Figure 5:**
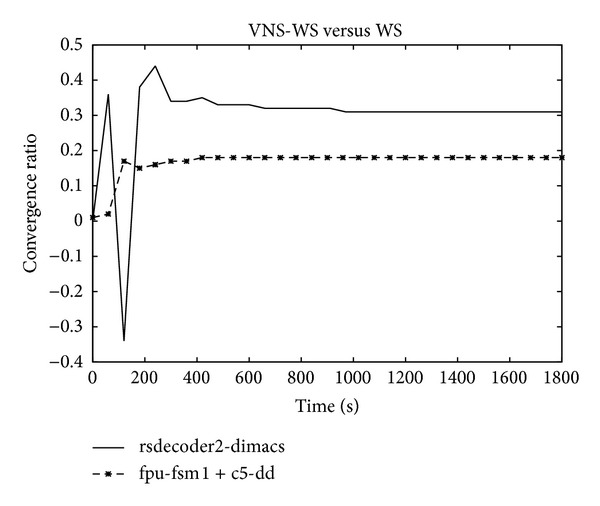
Convergence speed.

**Figure 6:**
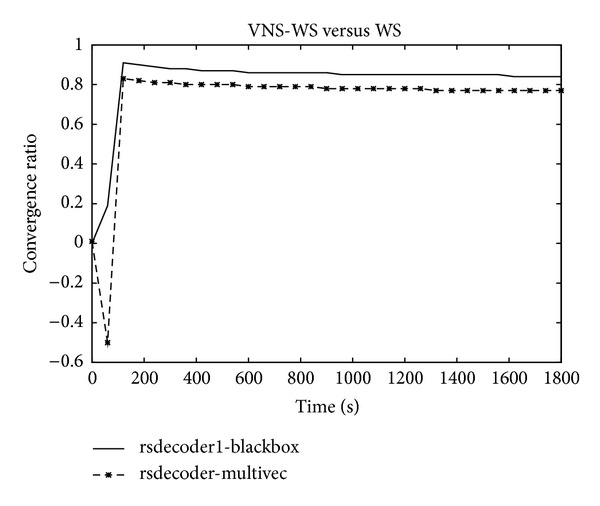
Convergence speed.

**Figure 7:**
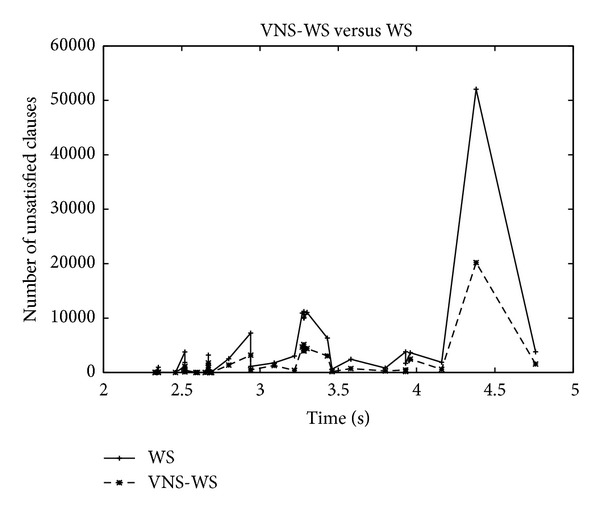
WS versus VNS-WS: clause to variable ratio.

Figures 5 and 6 show the convergence speed behavior expressed as the ratio between the mean of unsatisfied clauses of the two algorithms as a function of time. A negative value demonstrates the superiority of WS while a positive value confirms the opposite. For some instances, WS exhibits a better convergence speed during the early stage of the search before the ratio turns in favor of VNS-WS which starts demonstrating its dominance as the search continues. The asymptotic performance offered by VNS-WS is impressive and dramatically improves on WS. In some cases, the difference in the convergence speed reaches 20% during the first seconds and maintains this level during the whole search process as expressed in the right plot of Figure 5. However, on other cases, the difference continues to increase as the search progresses and gets as high as 93% as shown in Figure 6. The plot depicted in Figure 7 shows the number of unsatisfied clauses as a function of the clause to variable ratio. The first thing to notice is that as the ratio of clauses to variables increases, the number of unsatisfied clauses produced by VNS-WS remains lower while not showing a substantial variation compared to WS. The second thing is the existence of a crossover point at which the difference in the solution quality between the two algorithms is the highest. This turning point occurs at 4.5 and might represent the set of instances that are harder to solve.

### 5.3. Comparison of VNS-WS with Other Algorithms

Tables 1, 2, and 3 compare VNS-WS with three state-of-art algorithms (WS, Walksat with weights W-w, and variable weighting scheme VW2) using the package UCBSAT [42]. W-w and VW2 have proven to be very effective giving the best known results on some industrial benchmarks [43]. The first and second columns show the number of variables and clauses for the instance input. The last four columns show the number of unsatisfied clauses produced by each method. VNS-WS gave the better results than W-w and VW2 in 38 cases out of 44. When compared to VW2, the improvement ranges from 64% to 99% and from 29% to 99% when compared to W-w. Similar results were observed in 6 cases and beaten in one case by W-w. The comparison against WS shows that VNS-WS outperforms WS in 39 cases with an improvement ranging from 28% to 92% while similar results were observed in the remaining 5 cases.  Table 4 compares VNS-WS with highly efficient solvers CCLS [35] and Optimax which is a modified version of glucose SAT solver [44] ranked 1st at the 2011 SAT competition. CCLS won four categories of the incomplete algorithms track of Max-SAT Evaluation 2013. The instances used in the benchmark belong to random and crafted categories used at SAT2013 competition. VNS-WS gave similar quality results in 20 cases out of 27. However the time of CCLS ranges from 10% to 96% of the time of VNS-WS except in one case (s3v80-900-2) where VNS-WS was 39% faster compared to CCLS. In the remaining cases where VNS-WS was beaten, the difference in quality ranges from 2% to 11%. Another interesting remark to mention is that the time required by VNS-WS does vary significantly depending on the problem instance while the variations observed with CCLS remain very low. The comparison between Optimax and VNS-WS shows that Optimax converges very fast at the expense of delivering solutions of poor quality compared to VNS-WS. VNS-WS was capable of delivering solutions of better quality than Optimax in all the cases and the improvement ranges from 13% to 66%.

## 6. Conclusions and Future Research

In this work, a variable neighborhood search combined with Walksat (VNS-WS) for the maximum satisfiability problem is introduced. VNS-WS follows a simple principle that is based on systematic changes of neighborhood within the search. The set of neighborhoods proposed in this paper can easily be incorporated into any local search used for MAX-SAT. Starting the search from the largest neighborhood and moving systematically towards the smallest neighborhood is a better strategy to get a better heuristic. Thus, in order to get a comprehensive picture of the new algorithms performance, a set of large industrial instances is used. The results indicate that the proposed variable neighborhood strategy can enhance the convergence behavior of the Walksat algorithm. It appears clearly from the results that the performance of both WS and VNS-WS is fairly close with a slight edge in favor of VNS-WS for small problems. However, for larger problems, VNS-WS can find excellent solutions compared to those of WS at a faster convergence rate. The difference lies between 30% and 93%. The larger the problem, the larger the size of the neighborhood needed, and consequently the more efficient the WS at different neighborhoods. The results have shown that VNS-WS consistently delivers better solutions than Optimax while requiring the least amount of time. When compared to CCLS, VNS-WS was capable of providing similar results in 74% of the studied cases; however, the time invested is several orders of magnitude slower than CCLS. The author aims at submitting this solver for the next MAX-SAT competition after having improved its performance. For the time being, further work is mainly conducted on improving the solution quality of VNS-WS. In particular, during the construction of the different neighborhoods, the random merging scheme does not exploit the information structure of the problem. The author believes that VNS-WS might benefit from further research into merging strategies used to construct the neighborhoods. A better strategy would be to construct the different neighborhoods based on merging variables by exploiting the number of clauses they have in common rather randomly.

## Figures and Tables

**Algorithm 1:**
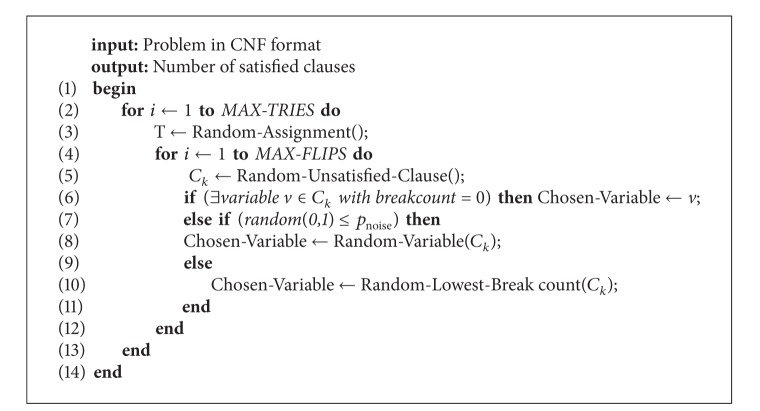
Walksat algorithm.

**Algorithm 2:**
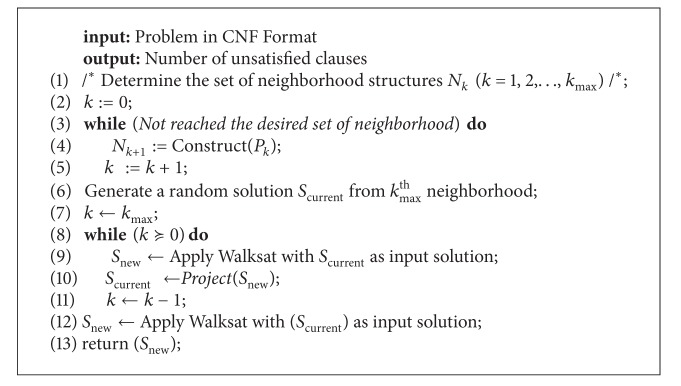
VNS-WS.

**Table 1:** SAT2013 industrial benchmarks: comparison among VNS-WS, WS, W-w, and VW2.

Instances	Instance input		Unsatisfied clauses			
	Var	Cls	WS	W-w	VW2	VNS-WS
diverders11.dimacs.filtered	45552	162982	2434	2101	4169	715
fpu8-problem.dimacs24.filtered	160232	548848	6328	6306	11290	3043
fpu-fsm1-problem.dimacs15.filtered	160200	548843	6125	6213	11855	3055
i2c-master2.dimacs.filtered	63816	221320	615	590	2037	161
b14-opt-bug2-vec1-gate-0.dimacs	130328	402707	1763	1749	5985	1279
b15-bug-fourvec-gate-0.dimacs	581064	1712690	7241	7596	22082	3184
b15-bug-onevec-gate-0.dimacs	121836	359040	1122	1244	4094	493
c1-DD-s3-f1-e2-v1-bug-fourvec-gate-0.dimacs	391897	989885	955	1317	4966	60
c1-DD-s3-f1-e2-v1-bug-onevec-gate-0.dimacs	102234	258294	62	191	592	2
c2-DD-s3-f1-e2-v1-bug-onevec-gate-0.dimacs	84525	236942	2537	2634	3699	1362
c3-DD-s3-f1-e1-v1-bug-fourvec-gate-0.dimacs	33540	86944	4	4	4	4
c3-DD-s3-f1-e1-v1-bug-onevec-gate-0.dimacs	8358	21736	1	1	1	1
c4-DD-s3-f1-e1-v1-bug-gate-0.dimacs	797728	2011216	3761	5714	15798	1129
c4-DD-s3-f1-e2-v1-bug-fourvec-gate-0.dimacs	448465	1130672	1834	1993	7772	516
c4-DD-s3-f1-e2-v1-bug-onevec-gate-0.dimacs	131548	331754	439	482	1899	105

**Table 2:** SAT2013 industrial benchmarks: comparison among VNS-WS, WS, W-w, and VW2.

Instances	Instance input		Unsatisfied clauses			
	Var	Cls	WS	W-w	VW2	VNS-WS
c5315-bug-gate-0.dimacs.seq.filtered	1880	5049	1	1	1	1
c5-DD-s3-f1-e1-v1-bug-fourvec-gate-0.dimacs	100472	270492	25	25	943	4
c5-DD-s3-f1-e1-v1-bug-gate-0.dimacs	200944	540984	97	153	2026	8
c5-DD-s3-f1-e1-v2-bug-gate-0.dimacs	200944	540984	59	101	1873	8
c5-DD-s3-f1-e1-v1-bug-onevec-gate-0.dimacs	25118	67623	11	11	65	1
c6288-bug-gate-0.dimacs.seq.filtered	3462	9285	14	16	63	1
c6-DD-s3-f1-e1-v1-bug-fourvec-gate-0.dimacs	170019	454050	1668	1644	4900	921
c6-DD-s3-f1-e1-v1-bug-gate-0.dimacs	298058	795900	3188	3332	9340	1752
c6-DD-s3-f1-e1-v1-bug-onevec-gate-0.dimacs	44079	117720	277	302	986	175
c6-DD-s3-f1-e2-v1-bug-fourvec-gate-0.dimacs	170019	454050	1645	1705	5066	919
c7552-bug-gate-0.dimacs.seq.filtered	2640	7008	1	1	1	1
divider-problem.dimacs-11.filtered	215964	709377	9992	10090	15889	3978
divider-problem.dimacs12.filtered	229482	751921	10914	10844	17297	4667
divider-problem.dimacs1.filtered	215676	708801	10181	10308	16102	4295
divider-problem.dimacs2.filtered	228874	750705	10688	11180	17319	4392

**Table 3:** SAT2013 industrial benchmarks: comparison among VNS-WS, WS, W-w, and VW2.

Instances	Instance input		Unsatisfied clauses			
	Var	Cls	WS	W-w	VW2	VNS-WS
divider-problem.dimacs3.filtered	216900	711249	10054	10727	16249	3972
divider-problem.dimacs4.filtered	225340	743637	11030	11536	17240	4406
divider-problem.dimacs5.filtered	228874	750705	11194	11929	17459	5139
mot-comb1-red-gate-0.dimacs.seq.filtered	2159	5326	1	1	1	1
mrisc-mem2wire1.dimacs.filtered	168960	641598	823	998	8191	293
rsdecoder2.dimacs.filtered	415480	1632526	3799	4811	24221	461
rsdecoder-fsm1.dimacs.filtered	238290	936006	1703	1906	12689	179
rsdecoder-problem.dimacs-34.filtered	226040	728516	3016	3880	11408	386
s15850-bug-fourvec-gate-0.dimacs.seq.filtered	88544	206252	62	84	176	21
s15850-bug-onevec-gate-0.dimacs.seq.filtered	22136	51563	3	1	5	2
SM-AS-TOP-buggy1.dimacs.filtered	145900	694438	3791	4023	11888	1549
SM-MAIN-MEM-buggy1.dimacs.filtered	870975	3812147	45360	52045	97604	20196
SM-RX-TOP.dimacs.filtered	235456	934091	3645	3528	13487	2456
spi2.dimacs.filtered	124260	515813	1872	1985	9993	581

**Table 4:** Comparing VNS-WS with CCLS and Optimax.

Instance	CCLS		Optimax		VNS-WS	
	Quality	Time	Quality	Time	Quality	Time
brock400-1	255	0.38	340	0.08	255	15.02
brock400-2	252	0.84	310	0.08	252	24.02
brock400-3	238	1.27	278	0.08	238	9.05
brock400-4	249	0.73	374	0.08	249	4.05
brock800-1	205	0.95	273	0.09	205	1.05
brock800-2	207	0.97	270	0.09	207	3.08
brock800-3	203	0.47	315	0.07	203	10.01
brock800-4	200	0.32	310	0.13	200	4.07
hamming10-2	400	0.09	532	0.08	400	1.04
hamming10-4	319	0.42	341	0.09	319	64.03
hamming6-2	832	1.18	1100	0.13	843	55.01
hamming6-4	192	1.00	312	0.13	192	1.06
hamming8-2	441	0.12	551	0.13	441	1.05
s2v120c1600-8	240	2.55	289	0.09	251	118
s2v140c1200-3	155	2.97	195	0.08	165	107
s2v140c1300-4	164	2.34	208	0.10	181	74
s2v140c1400-3	193	3.38	239	0.12	206	121
s2v140c1500-5	205	2.111	248	0.13	218	145
s3v80c600-5	12	1.02	16	32.61	12	1.06
s3v80c700-6	18	1.56	26	4.28	18	1.06
s3v80c800-2	32	1.24	59	0.12	32	48.02
s3v80c900-1	35	1.76	73	0.12	35	25.02
s3v80c900-10	35	1.60	59	0.11	35	33.07
s3v80c900-2	37	1.71	64	0.08	37	1.06
t5pm3-7777.spn	78	1.46	120	0.10	78	23.05
t6pm3-8888.spn	136	3.30	222	0.09	144	36.02
t7pm3-9999.spn	209	4.36	343	0.11	233	87
